# The “Eat Less Meat” one-month challenge: a randomized controlled trial of a meat reduction pledge intervention among French university students

**DOI:** 10.1186/s12966-025-01831-7

**Published:** 2025-10-27

**Authors:** Lucile Marty, Manon Biehlmann, Anne Louveau, Delphine Poquet, Eric Robinson

**Affiliations:** 1 Université Bourgogne Europe, Institut Agro, CNRS, INRAE, UMR CSGA, Dijon, 21000 France; 2 Université Paris Cité Et Université Sorbonne Paris Nord, Inserm, INRAE, Centre de Recherche en Épidémiologie Et StatistiqueS (CRESS), Villejuif Cedex, 94807 France; 3 Department of Psychology, University of Liverpool, Liverpool, L69 3GF UK

**Keywords:** Diet, Young adults, Intervention, Sustainability, Plant-based, Motivation

## Abstract

**Background:**

Encouraging the shift towards more plant-based diets in new generations is one of the major current challenges to preserve population and planetary health. Personal pledges to reduce meat consumption could motivate behaviour change, but have received limited scientific testing. We examined the effect of a “Eat Less Meat” one-month challenge on immediate and long-term meat consumption of university students.

**Methods:**

In January 2023, 366 university students (21 ± 3.2 years old) consented to participate in the “Eat Less Meat” one-month challenge and were randomized to the intervention group (*n* = 187, challenge in February 2023) or the wait list control group (*n* = 179, challenge in June 2023). Neither participants nor investigators were masked to group assignment. Participants chose between three meat reduction objectives: consuming meat 0, 3, or 6 times a week. They received a meat-free recipe book and followed an Instagram account where motivational information was posted daily during one month. All the participants completed a food frequency questionnaire in January (T0, before), February (T1, during), and May 2023 (T2, three months after the challenge) and data on meat consumption were analysed using linear mixed models.

**Results:**

The participants in the “Eat less meat” one-month challenge reduced their meat consumption by -67 g/day (95% CI [-82; -53]) during the challenge and by -50 g/day (95% CI [-68; -31]) three months later. The decrease was greater than in the control group by -34 g/day (95% CI [-55; -14]) during the challenge, but there was no significant difference between intervention and control group at three months follow-up.

**Conclusions:**

The “Eat Less Meat” one-month challenge may be a promising strategy to drive short-term reductions in meat consumption and further work to improve longer-term effectiveness is now warranted.

**Trial registration:**

The trial was pre-registered prior to data collection at Clinicaltrials.gov (NCT05752786).

**Supplementary Information:**

The online version contains supplementary material available at 10.1186/s12966-025-01831-7.

## Background

Encouraging the shift towards sustainable diets in young adults is one of the major current challenges to preserve population and planetary health. Current food systems are responsible for millions of deaths each year and for a third of global anthropogenic greenhouse gas emissions [1, 2]. In this context, the Food and Agriculture Organization and World Health Organization have outlined targets for sustainable diets defined as promoting individuals' health and well-being, having low environmental impact, being accessible to all and culturally acceptable [3]. In terms of what to eat, sustainable diets particularly need to be reduced in meat consumption which has negative impacts on human health and the environment [4].

The transition to post-secondary education involves a significant life change and is accompanied with the adoption of less healthy dietary behaviours [5]. University students usually show a decrease in diet quality when leaving the family home, with adoption of diets that do not align with sustainable diets guidelines [6]. Yet, the developmental stage of university students, i.e., navigating new eating independence, establishing identity, building lifelong habits, offers unique opportunities to promote new dietary behaviours that will persist into later life [7]. University students are indeed particularly more willing to adopt changes in their eating behaviours and are more environmentally conscious than older generations [8] which makes this population an ideal target for interventions aiming at improving the sustainability of diets.

Motivation is a central component of most theories of behaviour [9] and is defined as the brain processes that drive behaviour, including habitual processes (automatic motivation) and analytical decision-making (reflective motivation) [10]. Motivation has been repeatedly shown to be associated with consumption of more sustainable diets in cross-sectional studies [11–13], but is overlooked in real-world intervention strategies that are mainly based on education (e.g., labelling) or on environmental restructuring (e.g., food choice architecture) [14–16]. A recent study reviewed the available evidence on university-implemented meat reduction interventions and reported five strategies: promotional messaging, pricing incentives, dining area layout changes, menu labelling changes and menu offering changes [17]. Most were successful in reducing the amount of meat consumed but only within university dining environments, without considering potential compensatory effects (e.g., eating more meat later in the day). We argue that motivation targeted interventions have the potential to change overall diet. There are various behavioural change techniques able to enhance motivation to perform a behaviour [10]. Goal setting over one month may promote the repetition of the target behaviour and then help to form new habits thus targeting automatic motivation [18]. Persuasive messages may progressively contribute to a more analytical decision-making based on the expected outcomes of the behaviour thus targeting reflective motivation [19]. Short-term pledge-based challenges, such as abstinence from alcohol (‘Dry January’) [20] are motivation-based interventions and may be effective in reducing alcohol consumption, but their potential in changing meat consumption has received limited scientific testing.

The “Eat Less Meat” one-month challenge was conceived as a motivation-based behavioural intervention to reduce meat consumption in university students by encouraging students to voluntarily pledge to reduce their meat consumption. More specifically, the challenge aims (i) to target *automatic motivation* by weakening meat consumption habits and (ii) to target *reflective motivation* by enhancing food choice motives associated to lower meat consumption. The aim of the present study was to evaluate the effects of the challenge in a randomized controlled trial both during and three months after. We hypothesized that participating in the challenge would decrease meat consumption (H1, primary outcome: daily meat intake in grams), increased nutritional quality and decrease environmental impact of the diets (H2, secondary outcomes), weaken meat consumption habits (H3, secondary outcome), and increase health, ethics, and animal welfare motivations for food choices (H4, secondary outcomes).

## Methods

### Study design

This study was a parallel two-arm randomized controlled trial with repeated online measures pre- (T0), during- (T1), and post-intervention (T2, three months after) to evaluate a one-month motivation-based intervention to reduce meat consumption. The study was conducted in Dijon, France, among university students. Data collection started in January 2023 and ended in June 2023. The study was approved by an ethics committee (N°22–155, CEEI/IRB Inserm) and the trial was pre-registered prior to data collection at Clinicaltrials.gov (NCT05752786). The full study protocol is available on the OSF webpage of the project: https://osf.io/3gts8/.

### Participants

University students were recruited in January 2023 through emails, social media, flyers, and on-campus posters with a link or QR-code to an online eligibility questionnaire. University students were eligible if they were aged 18 or above, registered in a Bourgogne-Franche-Comté university, fluent in French, had an email address and Instagram account, were not pescatarian, vegetarian nor vegan, were not pregnant or breastfeeding, and did not have iron deficiency or a body mass index below 18.5. All eligibility criteria were self-reported. Informed consent was obtained from all the participants through an online form and they received a 10€ voucher in return for questionnaires completion (30€ in total).

### Randomisation and masking

**Fig. 1:**
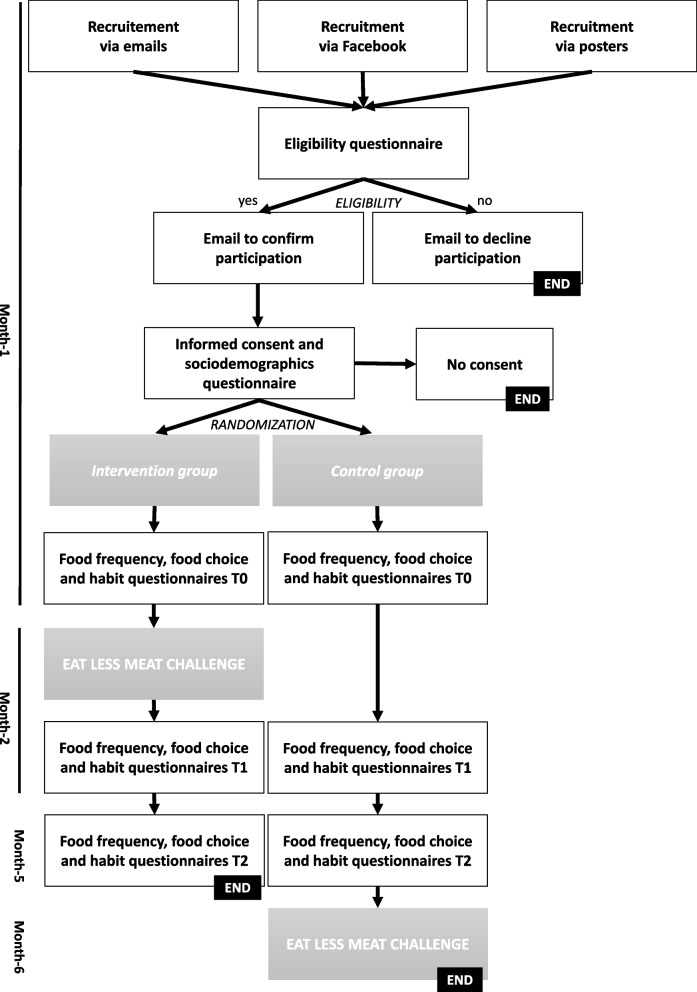
Study framework

All the participants were recruited to take part in the “Eat Less Meat” one-month challenge to ensure similar initial motivational profile towards meat reduction. After providing their informed consent, they were randomized sequentially into the February challenge (intervention group) or June challenge (wait list control group) following a 1:1 randomisation sequence generated before recruitment using the Random Allocation software [21]. Participants had the option to take part with friends and groups of friends were assigned to the same experimental group (cluster randomization). Neither participants nor investigators were masked to group assignment. Participants in the control group took part in the challenge three months after the participants in the intervention group (wait list control group). Participants in both groups completed the online measures at the exact same time meaning that pre- (January, T0), during- (February, T1), and post-intervention (May, T2) measures all took place before the control group had started the challenge, in June. The study framework is summarized Fig. 1.

### Procedures

After providing informed consent, participants were asked in an online questionnaire to declare their usual meat consumption frequency per week (without indication of what was defined as a serving) and to choose a personalized level for the “Eat less meat” one-month challenge: consuming (i) no meat, (ii) meat 3 times a week, (iii) meat 6 times a week; which had to be lower than their usual meat consumption frequency. The meat category includes all red meat and poultry but not fish or other animal-sourced food products (eggs, dairy, etc.). This pledge targeted *automatic motivation* for meat consumption by attempting to disrupt habits through the repetition of meat avoidance during one month.

The first day of the challenge, the participants received by email a cookbook of ten easy-to-prepare and cheap meat-free recipes and a starter kit with advice and motivational tips. During the month of the challenge, they received an email each week (four in total) with more advice and motivational tips. They were also asked to follow the Instagram account of the “Eat less meat” one-month challenge (reduirelaviande_fev23, in French) where during one month one publication was posted every day except on Sundays, engaging stories requiring interactions were posted regularly, participants could ask their questions to the research team, and participants could communicate. The materials are available (in French) on the Open Science Framework webpage of the project (cookbook, starter kit, emails, Instagram publications): https://osf.io/3gts8/.

The topics covered in the online materials were selected following an analysis of the target behaviour based on literature reviews on meat consumption reduction (e.g., [13, 14]) and using the Capability-Opportunity-Motivation-Behaviour (COM-B) framework [10]. We identified three main themes: (i) increasing awareness regarding the impacts of dietary habits on health, environment and animal welfare (*reflective motivation*), (ii) providing ideas and tips for healthy and tasty meat-free cooking (*capability*), (iii) avoiding social and contextual pressure to eat meat (*opportunity*). We also held two discussion groups with university students from Dijon (*n* = 15) with the aims to: (i) identify barriers and levers for meat consumption reduction that we may have missed or would have been specific to the local context, (ii) gain insights regarding how to recruit participants for the “Eat less meat” one-month challenge, (iii) get feedback on the design of the online materials. The discussion groups confirmed the relevance of the three main themes previously identified and helped to precisely define which information would be the most useful for university students trying to reduce their meat consumption in the local context.

The online materials targeted both *reflective motivation* (directly) by attempting to enhance health, environment and animal welfare concerns and *automatic motivation* (indirectly through capability and opportunity) by attempting to alleviate barriers to reduce meat consumption and thus increasing participants’ commitment to the challenge.

### Measures

At inclusion, the participants were asked to complete an online sociodemographic questionnaire and to self-report their age, gender (man, woman or other), scholarship status (yes: in receipt of financial support due to being a lower socioeconomic position student, or no), weight and height, and type of diet (omnivorous defined as “I usually eat animal products” or flexitarian defined as “I usually limit my consumption of animal products”). At three time points, pre- (T0), during- (T1), and post-intervention (T2), the participants were asked about their food consumption, food choice motives, and meat consumption habit for the previous month, meaning for the month before, the month during, and the third month after the challenge of the intervention group. All the measures were completed online before the control group had started the challenge, see Fig. 1.

The food frequency questionnaire has previously been validated [22] and included 109 foods, 12 non-alcoholic drinks and four alcoholic drinks with frequency assessed by a six-item scale: ‘Never’, ‘Between once and three times a month’, ‘Once a week’, ‘Between two and five times a week’, ‘Once a day or almost’, ‘Several times a day’. Frequency was defined as the number of eating occasions without indication of what was defined as a serving. Instead, participants estimated their usual portion size on one eating occasion for 71 food items and the 12 non-alcoholic drinks using photos on a five-point scale derived from the SU.VI.MAX portion book [23]. For the 38 remaining food items, the midportion in the SU.VI.MAX portion book was automatically assigned. Among the 109 food items, 13 were meat-based items all paired with a self-declared usual portion size. Consumption frequencies of each item were transformed into daily frequencies and daily intake was calculated by multiplying the daily frequency by the usual portion size.

The strength of meat consumption habit was assessed using the validated Self-Report Habit questionnaire [24] including 12 items accompanied by a six-point response scale anchored by ‘Strongly disagree’ and ‘Strongly agree’ from 1 to 6 with high values indicating strong habits.

The food choice motives questionnaire was the adapted and validated French version of the Food Choice Questionnaire [25, 26] previously used in student population [27]. It includes 30 items and ten subscales: health, mood, convenience, sensory appeal, natural content, price, weight control, familiarity, ethics, and animal welfare. Answers to each item ranged from 1 to 4: 1 = ‘Not at all important’; 2 = ‘A little important’; 3 = ‘Moderately important’; 4 = ‘Very important’.

### Outcomes

All the outcomes were computed for each participant at three time points: pre- (T0), during- (T1), and post-intervention (T2).

#### Primary outcome

The primary outcome was daily meat intake (in grams) calculated by summing daily intake for the 13 items of the food frequency questionnaire that fall into the meat category (i.e., beef patties, beef, pork, veal, lamb, poultry, liver, other offal, salami, mortadella, ham and sausages) and the meat content from the mixed dishes items estimated based on university cafeterias recipes database.

#### Secondary outcomes

We estimated the nutritional quality based on the food frequency questionnaire data as the adherence to the French dietary guidelines evaluated by the simplified PNNS-GS2 [28]. The sPNNS-GS2 calculation considers negative components (less healthy food groups such as red meat, processed meat, sugary foods, sweet-tasting beverages, alcoholic beverages, and salt) and positive components (healthier food groups such as fruits and vegetables, nuts, legumes, whole-grain food, milk and dairy products, and fish and seafood). sPNNS-GS2 was calculated excluding added fat recommendation as this component was not measured in the food frequency questionnaire (range: −17 to 11.5).

We estimated the environmental impact based on the food frequency questionnaire as the greenhouse gas emissions (GHGE) derived from the French food environmental impact database Agribalyse 3.0 drawn up by the French Agency for Ecological Transition that includes values for 2480 food products based on life cycle analyses [29]. All the items of the food frequency questionnaire were associated to all the corresponding food products from Agribalyse 3.0. GHGE of each item of the food frequency questionnaire were calculated as the average GHGE of all the food products from Agribalyse 3.0 associated a given item. GHGE of participants’ daily diets were calculated by multiplying the daily intake of each food item by its associated GHGE per kg and summing across all the items.

Meat consumption habit was estimated by the Self-Report Habit Index (SRHI) computed by averaging ratings for all the individual items in the Self-Report Habit questionnaire (range: 1 to 6). Health, environmental, and animal welfare concerns scores were calculated by averaging ratings for individual items in the health, ethics, and animal welfare subscales of the food choice questionnaire, respectively (range: 1 to 4).

#### Non-pre-registered outcome

Individual energy intake was calculated by multiplying the daily intake of each food item by their energy density retrieved from the SU.VI.MAX nutrient composition database [30].

### Statistical analyses

We followed a pre-registered analysis plan available on the Open Science Framework webpage of the project: https://osf.io/3gts8/. We used linear mixed models to investigate whether changes in primary and secondary outcomes pre- (T0), during- (T1), and post-intervention (T2) differed significantly between participants in the intervention and the control group. The outcomes based on the food frequency questionnaire (meat consumption, nutritional quality, and environmental impact) were coded as missing for participants considered as outliers for energy intake at a given time point, defined as participants in the first and last percentile of energy intake at a given time point. For supplementary analyses, dietary outcomes were also coded as missing for participants reporting implausible energy intake at a given time point, defined as being outside of the following ranges: 500–3500 kcal for women and 800–4200 kcal for men as recommended for food frequency questionnaire data [31]. BMI was coded as missing for implausible values. We reported the number of missing (or implausible values) for each outcome at each time point.

The main analyses were conducted as intention to treat based on observed data and sensitivity analyses were conducted using multiple imputation, first on missing values only and then on missing and implausible values. Multiple imputation (*n* = 100) was performed using fully conditional specification separately for control and intervention groups to allow for different associations between covariates and outcomes in each group. The variables included in the imputation models were outcomes at T0, outcomes at T1, outcomes at T2, age, gender, BMI, and the challenge level.

We ran linear mixed models with meat consumption (H1), nutritional quality (H2), environmental impact (H2), Self-Report Habit Index (H3), health motives (H4), ethics motives (H4), animal welfare motives (H4), and energy intake (not pre-registered) as dependent variables, time (T0, T1 and T0, T2), group (intervention, control), time*group interaction as fixed effects and both participant and cluster (friendship group allocated to the same experimental group) as random effects. In error we did not specify using a clustered approach in our pre-registration. The models were adjusted for gender, age, and BMI in order to control for potential confounding effects. We estimated adjusted mean differences between T1 and T0, and between T2 and T0 for the intervention and the control groups separately, and differences in differences between the two groups.

In exploratory analyses we ran two additional models on the intervention group only to investigate whether the level of the challenge (i.e., (i) no meat, (ii) meat 3 times a week, (iii) meat 6 times a week) differentially influenced meat consumption reduction (observed data). The first linear mixed model was run with meat consumption as dependent variable, time (T0, T1 and T0, T2), level (i, ii, iii), time*level interaction as fixed effects and participant as random effect. We estimated adjusted mean differences between T1 and T0, and between T2 and T0 for each level, and differences in differences with level (i) as reference. In the second linear mixed model, we substituted the level of the challenge by the difference between baseline meat consumption frequency (in times per week) and the level of the challenge, i.e. the meat reduction objective (continuous variable).

All statistical analyses were performed using SAS version 9.4 (SAS Institute, Inc., 2012 SAS® 9.4. Cary, NC). The proc mixed was used for mixed models, the proc MI and proc mianalyze for multiple imputation. The level of significance was set at *p* < 0.05 for all pre-registered analyses.

### Sample size

Sample size was determined based on a power analysis (GPower v3.1) to detect a small effect size of f = 0.10 for a within-between interaction (i.e., time*group) in a mixed model. A total sample size of 164 participants was necessary to reach 0.80 power at 0.05 alpha under individual randomization. Considering an anticipated attrition rate of 15–20% and a small number of participants taking part with friends, we aimed to recruit a minimum *n* = 200, whilst allowing all participants registering interest to participate. Registrations for the challenge closed on the day before the challenge started for the intervention group.

## Results

**Fig. 2:**
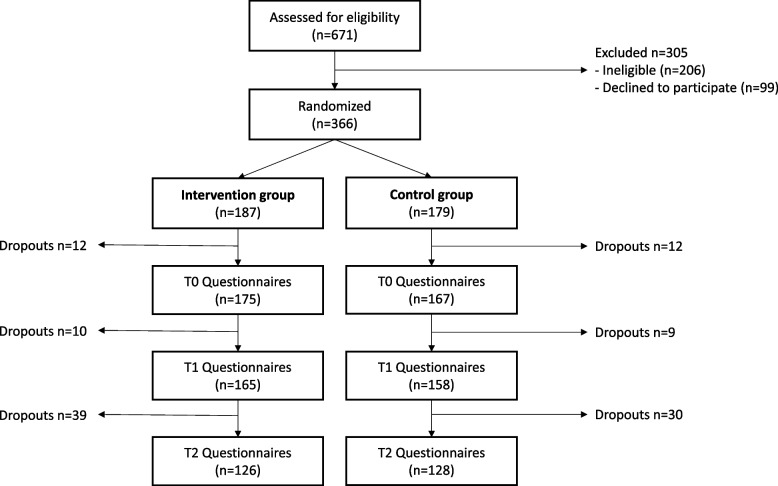
Trial flow chart

From January 3rd, 2023 to January 31 st, 2023, 671 people completed the online eligibility questionnaire and the 465 who were eligible received the informed consent form by email. Reasons for ineligibility were: BMI < 18.5 (*n* = 55), vegetarian (*n* = 52), not in a Bourgogne-Franche-Comté university (*n* = 37), no Instagram account (*n* = 22), not fluent in French (*n* = 17), not willing to reduce meat consumption (*n* = 17), age < 18 (*n* = 6). Among the eligible participants, 366 completed the online consent form, chose their personalized level for the challenge, completed the sociodemographic questionnaire and were randomized between the intervention and control group. The attrition rate was 7% at T0 (*n* = 342), 12% at T1 (*n* = 323), and 31% at T2 (*n* = 254) (Fig. 2). At inclusion, 128 participants intended to participate with friends (3 clusters of 5, 1 cluster of 4, 11 clusters of 3, 38 clusters of 2) and 238 alone, resulting in a total of 291 clusters. The number of clusters decreased at T0 (n_c_ = 272), T1 (n_c_ = 261) and T2 (n_c_ = 212) due to attrition. Although attrition rate and clustered participation was higher than expected, the number of individual participants and the number of clusters itself remained higher than the target sample size of 164 at each time point.

Enrolled participants were mostly women (74%) and a third declared following a flexitarian diet (i.e., limiting consumption of animal-sourced food). Half of the participants chose the “3 times a week” level for the challenge, with on average a meat reduction goal of −3 to −4 times per week compared to baseline, which was similar in both groups (Table 1).

**Table 1:** Participants’ characteristics overall and by group, from the sociodemographic questionnaire

	**All** (*n* = 366)	**Intervention** (*n* = 187)	**Control** (*n* = 179)
Age, years, mean (SD)	21.6 (3.2)	22.0 (3.6)	21.3 (2.7)
Gender, women, *n* (%)	272 (74)	145 (71)	127 (77)
Scholarship^1^, yes, *n* (%)	139 (38)	73 (39)	66 (37)
BMI, kg/m^2^, mean (SD)	22.6 (3.5)	22.6 (3.4)	22.8 (3.7)
*Missing BMI, n*	10	6	4
Declared diet, *n* (%)			
Omnivorous	241 (66)	137 (73)	104 (58)
Flexitarian	125 (34)	50 (27)	75 (42)
Declared meat consumption, frequency/week, mean (SD)	6.5 (2.9)	6.8 (2.8)	6.1 (3.0)
Challenge level, *n* (%)			
No meat	94 (26)	35 (19)	59 (33)
3 times a week	199 (54)	113 (60)	86 (48)
6 times a week	73 (20)	39 (21)	34 (19)
Meat reduction objective, frequency/week, mean (SD)	−3.7 (1.9)	−3.8 (1.9)	−3.5 (1.8)

^1^In receipt of financial support due to being a lower socioeconomic position student

Baseline values for all primary and secondary outcomes overall and by group are presented Table 2 and adjusted means comparisons from linear mixed models are presented Table 3.

**Table 2:** Baseline values as means (SD), all variables from T0 questionnaire

	**All** (*n* = 342)	**Intervention** (*n* = 175)	**Control** (*n* = 167)
Meat consumption^1^, g/day	114 (114)	115 (102)	113 (126)
sPNNS-GS2^1^, range [−17 to 11.5]	0.4 (2.8)	0.7 (2.8)	0.1 (2.8)
GHGE^1^, kgCO_2_eq/day	5.4 (3.3)	5.4 (2.9)	5.4 (3.7)
Energy^1^, kcal/day	2056 (893)	2019 (827)	2095 (958)
SRHI, range [1; 6]	3.5 (1.1)	3.6 (1.1)	3.3 (1.1)
Health motives, range [1; 4]	2.6 (0.7)	2.7 (0.7)	2.6 (0.8)
Ethics motives, range [1; 4]	2.7 (0.8)	2.7 (0.8)	2.7 (0.8)
Animal welfare motives, range [1; 4]	2.7 (0.8)	2.7 (0.8)	2.7 (0.8)

*GHGE* greenhouse gas emissions, *SRHI* Self-Report Habit Index

^1^*n* = 336 (intervention: *n* = 173, control: *n* = 163) after exclusion of outliers for energy intake (< 1 st or > 99th percentile)

**Table 3:** Adjusted means comparisons from linear mixed models (main analyses, *n* = 342)

	**Diff (T1-T0)** **95% CI**	**Diff (T2-T0)** **95% CI**	**Diff**_**int**_**-Diff**_**ctr**_ **(T1-T0)** **95% CI**	**Diff**_**int**_**-Diff**_**ctr**_ **(T2-T0)** **95% CI**
Meat consumption, g/day				
Intervention	**−67 [−82; −53]**	**−50 [−68; −31]**	**−34 [−55; −14]**	−3.0 [−29; 23]
Control	**−33 [−48; −18]**	**−47 [−65; −28]**		
sPNNS-GS2, [−17 to 11.5]				
Intervention	**1.1 [0.7; 1.5]**	**0.7 [0.2; 1.1]**	0.2 [−0.3; 0.8]	−0.1 [−0.8; 0.5]
Control	**0.9 [0.5; 1.3]**	**0.8 [0.4; 1.3]**		
GHGE, kgCO_2_eq/day				
Intervention	**−1.4 [−1.8; −1.0]**	**−1.6 [−2.1; −1.2]**	−0.5 [−1.1; 0.1]	−0.3 [−1.0; 0.3]
Control	**−0.9 [−1.3; −0.5]**	**−1.3 [−1.8; −0.8]**		
Energy^1^, kcal/day				
Intervention	**−273 [−373; −174]**	**−430 [−565; −295]**	−14 [−156; 127]	−60 [−251; 130]
Control	**−259 [−360; −158]**	**−369 [−504; −235]**		
Self-Report Habit Index, [1; 6]				
Intervention	**−0.7 [−0.8; −0.6]**	**−0.8 [−1.0; −0.6]**	**−0.5 [−0.6; −0.3]**	**−0.4 [−0.7; −0.2]**
Control	**−0.2 [−0.3; −0.1]**	**−0.4 [−0.5; −0.2]**		
Health motives, [1; 4]				
Intervention	**0.1 [0.1; 0.2]**	**0.1 [0.1; 0.2]**	0.1 [−0.1; 0.2]	0.1 [−0.1; 0.2]
Control	0.1 [−0.1; 0.2]	0.1 [−0.1; 0.2]		
Ethics motives, [1; 4]				
Intervention	**0.1 [0.1; 0.2]**	0.1 [−0.1; 0.2]	0.1 [−0.1; 0.2]	0.1 [−0.1; 0.3]
Control	0.1 [−0.1; 0.1]	−0.1 [−0.1; 0.1]		
Animal welfare motives, [1; 4]				
Intervention	**0.2 [0.1; 0.3]**	**0.2 [0.1; 0.3]**	**0.2 [0.1; 0.3]**	**0.2 [0.1; 0.4]**
Control	0.1 [−0.1; 0.1]	0.1 [−0.1; 0.1]		

*CI* confidence interval, *GHGE* greenhouse gas emissions, *SRHI* Self-Report Habit Index. In bold: significant differences at alpha = 0·05

^1^Not pre-registered

The participants in the “Eat less meat” one-month challenge reduced their meat consumption by −67 g/day (95% CI [−82; −53]) during the month of the challenge compared to the month before the challenge, and by −50 g/day (95% CI [−68; −31]) three months after the challenge (Table 3). Relative to the participants in the control group who also significantly reduced their meat consumption, the participants in the intervention group reduced their meat consumption by −34 g/day (95% CI [−55; −14]) during the month of the challenge but the difference between the two groups was no longer significant three months after the challenge (−3.0 g/day (95% CI [−29; 23])) (Table 3).

The nutritional quality of the diets increased and the environmental impact decreased in both groups during and three months after the challenge, with no significant differences between the two groups (Table 3). Participants in the intervention group showed a greater reduction in meat consumption habits compared to control at both time points. In addition, participants in the intervention group had a greater increase in animal welfare motives than control at both time points. No other differences between intervention and control groups were observed for health motives or ethics motives (Table 3).

These results were confirmed when questionnaires with implausible values for energy intake were excluded except that differences in differences between the two groups became significant for GHGE, with a larger decrease in the intervention group both during and three months after the challenge (Supplementary material 1). In sensitivity analyses using multiple imputation on missing values only and on missing and implausible values for energy intake, results suggested that the effects of the challenge on primary and secondary outcomes were robust for the primary outcome and similar for secondary outcomes (Supplementary materials 2 and 3).

At inclusion, participants in the intervention group who pledged to eat meat 6 times a week during the challenge (*n* = 73) declared an average consumption of 10.5 (1.9) times a week (meat reduction target: −4.5 (1.9) times/week), those who pledged to eat meat 3 times a week (*n* = 199) declared an average consumption of 6.4 (1.8) times a week (meat reduction target: −3.4 (1.8)), those who pledged to eat no meat (*n* = 94) declared an average consumption of 3.6 (1.6) times a week (meat reduction target: −3.6 (1.6)).

Across the three levels, the participants reduced their meat consumption both during and three months after the challenge (Table 4). The participants who chose the “6 times a week” level for the challenge reduced their meat consumption by a further −52 g/day (95% CI [−56; −14]) during the month of the challenge compared to the participants who chose the “no meat” level, but the difference was no longer significant three months after the challenge (−28 g/day (95% CI [−75; 20])) (Table 4). The meat reduction size of the target was associated with meat intake reduction (−12 g/day, 95%IC [−19; −5.4]), but meat intake reduction three months after the challenge was not associated with the size of the meat reduction target (−4.7 g/day, 95%IC [−13; 3.8]).

**Table 4:** Adjusted means comparisons from linear mixed model (exploratory analyses, *n* = 175)

Meat consumption	Diff (T1-T0) 95% CI	Diff (T2-T0) 95% CI	Diff_6/3_-Diff_0_ (T1-T0) 95% CI	Diff_6/3_-Diff_0_ (T2-T0) 95% CI
Challenge level				
6 times a week	**−90 [−118; −63]**	**−69 [−100; −38]**	**−52 [−93; −11]**	−28 [−75; 20]
3 times a week	**−70 [−89; −52]**	**−46 [−66; −25]**	−32 [−67; 3.7]	−3.9 [−45; −38]
No meat	**−39 [−69; −8.4]**	**−42 [−78; −5.5]**		

In bold: significant differences at alpha = 0.05

## Discussion

Using a randomized controlled trial design, we evaluated the effects of the “Eat Less Meat” one-month challenge which involved participants pledging to reduce their meat intake. Participants in the intervention group reduced their meat consumption during and three months after the challenge compared to baseline. The reduction in the intervention group was significantly larger than the reduction observed in the wait list control group during the challenge, but not three months later.

The results were only partly in line with our first hypothesis, confirming short-term effects of the challenge on meat consumption, but not longer term effects. Both intervention and control groups showed the same decrease in meat consumption three months after the trial. We expected meat reduction during the challenge and three months after in the intervention group, but did not expect to observe such changes in the wait list control group. Contamination of the intervention in the control group is unlikely as the intervention content on social media was only accessible to registered participants in the intervention group. Close contact between participants in the control and intervention groups is also unlikely as participants were recruited from 20,000 students on campus and groups of friends were assigned to the same group. Behaviour change in control groups enrolled in randomized control trials is common [32]. In our case, the participants in the control group were also invited to take part to the “Eat Less Meat” one-month challenge but after the study completion (wait list control). We hypothesize that participants in the control group may have anticipated meat reduction as soon as they were enrolled in the study. To strengthen this hypothesis, we conducted a contextual analysis of other factors that could have explained meat reduction in university students during the time of study. In March 2023, we searched Google News with combinations of the following keywords: February 2023, sustainable food, vegetarian, meat, university students, Dijon, France. We also went through the Dijon city, Burgundy University and Burgundy Region websites and social medias. We found no increase in meat prices nor events that could have triggered meat reduction during this period. We hence suggest that being enrolled in a meat reduction trial led to behaviour change even though the actual intervention had not been delivered yet.

A recent systematic review investigated the effectiveness of interventions promoting sustainable dietary behaviour and identified 13 eligible trials [15]. Most of those studies used education as intervention function (11/13) and were randomized controlled trials (8/13) with follow-up ranging from one week to five months. Across the ten studies evaluating interventions on meat reduction, pre-post differences within the intervention groups varied from −0.7 to −3.7 servings per week, which is lower than the pre-post reduction of −4.7 servings per week (−67 g*7d/100 g) observed in the intervention group during the “Eat Less Meat” one-month challenge (considering 100 g-servings of meat). Across the four studies evaluating carbon footprint reduction, pre-post differences varied from −1.5 to −10.5 kgCO_2_eq per week thus also generally lower than the pre-post reduction of −9.8 kg CO_2_eq per week (−1.4 kg*7d) observed in the intervention group during the “Eat Less Meat” one-month challenge. However, it is important to note that we observed reductions in carbon footprint in the control group and the size of difference between intervention and control in the present study was not significant.

We found no differences between intervention and control groups for changes in nutritional quality and environmental impact of the diets, with both intervention and wait list control groups having increased nutritional quality and decreased environmental impact during the trial and three months after. The lack of effect on nutritional quality and environmental impact was surprising considering the literature on the positive association between vegetarian diets and nutritional quality and the negative association with greenhouse gas emissions [33–35]. We suggest that more guidance regarding healthy meat substitutions could have helped the participants to increase the nutritional quality and decrease the environmental impact of their diets while reducing meat consumption.

The fact that habits of consuming meat significantly decreased in the intervention group compared to the control group both during and three months after the challenge is a particularly promising result in a context where evidence for intervention strategies that trigger long-term meat reduction is scarce [13, 14]. This significant decrease in meat consumption habits was however quite small: −0.5 points during the challenge and −0.4 points three months after on a scale ranging from 1 to 6. A change in habits is needed to promote long-term behaviour change but can be difficult to achieve [36]. The Eat Less Meat challenge also enhanced animal welfare food choice motives (vs. control) which has been found to be important to adopt and keep a vegetarian diet [37]. This significant increase in animal welfare food choice motives was however quite small: + 0.2 points during and three months after the challenge on a scale ranging from 1 to 4. These results suggest that there may be longer-term beneficial effects of an “Eat Less Meat” one-month challenge, which now warrants further investigation including longer-term follow-up.

Universities may be ideal settings to promote the transition towards sustainable diets [38] and the “Eat Less Meat” one-month challenge may be a useful tool for universities wanting to lead on this transition. First, it actively involves the students by promoting action beyond solely delivering information. Second, it constitutes a good opportunity to investigate whether affordable, satisfying, and healthy plant-based options are available on campus and if not to initiate a change. Third, it may encourage university staff wanting to raise the issue of the (un)sustainability of the food systems with their students and colleagues. By empowering all the university stakeholders, we think that organizing a “Eat Less Meat” one-month challenge would have the potential to open a space to discuss and act in favour of more sustainable diets at the scale of a university campus.

The present study has several strengths and limitations. We used a randomized controlled trial design to evaluate the effects of a real-life challenge targeting meat reduction. The control condition was a wait list control and its inclusion allowed us to ensure a comparison group with similar initial motivation to reduce meat consumption. However, there was no participant engagement during the actual study period in which the intervention was delivered; an active control (i.e. participant engagement in activity) in future trials would provide more confidence in intervention effects. For the intervention group, we did not have control regarding participants’ exposure to what was posted on the Instagram account. We however asked them for feedback at the end of the challenge through an online questionnaire: among 165 respondents, 88% declared seeing content from the account on Instagram. Participants were not blinded as they knew when they were taking part in the challenge and data collection may thus have been prone to demand bias, i.e., participants changing their behaviour due to awareness of research hypotheses or study aims. Experimenters were responsible to send the intervention materials to the participants and were thus not blinded to the participants group assignment, which is a limitation. However, as there was no direct interaction with participants during data collection (online questionnaires only) and the analyses plan was pre-registered before data collection, bias is likely to be minimal. Attrition at follow-up was higher than expected (31%). However, analyses using multiple imputation on missing data produced the same results as main analyses.

Our findings are promising although some participants may have been more motivated by the challenge as it was administered by a research team and they had to complete food consumption questionnaires prone to social desirability bias thus increasing the effects of the challenge on meat reduction. Beyond measuring the target behaviour, we recorded participants whole diet and calculated both nutritional and environmental scores to actually assess the effects on diet sustainability. Diet was self-reported and this can be prone bias. Although food consumption was self-declared, we used a validated quantitative food frequency questionnaire and ran multiple sensitivity analyses to strengthen confidence in our results. An important limitation of the food frequency questionnaire we used is that it may not have fully captured the substitutions after meat reduction as no additional items have been included (e.g., vegan steaks, vegan sausages) apart from the meatless versions of the ready meals (e.g., meatless lasagne, meatless burger). Although this tool was originally validated in a pregnancy cohort, the 125 food items included were generic and not specific diet during pregnancy. As intervention strategy and interventional tools were developed for the student population, results may not generalise to other population subgroups.

## Conclusions

The “Eat Less Meat” one-month challenge may be a promising strategy to drive short-term reductions in meat consumption and further work to improve longer-term effectiveness is now warranted.

## Supplementary Information

Supplementary Material 1.

Supplementary Material 2.

Supplementary Material 3.

Supplementary Material 4.

## Data Availability

Anonymous individual participant data and a data dictionary defining each field are publicly available on the OSF webpage of the project: https://osf.io/3gts8/

## Abbreviations

COM-B: Capability-Opportunity-Motivation-Behaviour framework
GHGE: Greenhouse gas emissions
sPNNS-GS2: Simplified score of adherence to the French dietary guidelines
