# Supplementary material for: The “Eat Less Meat” one-month challenge: a randomized controlled trial of a meat reduction pledge intervention among French university students
Source: Int J Behav Nutr Phys Act. 2025 Oct 27;22:131. doi: 10.1186/s12966-025-01831-7 (PMC12557974; doi:10.1186/s12966-025-01831-7)
Supplement: Supplementary file 3 — Supplementary Material 3. [file 12966_2025_1831_MOESM3_ESM.docx]

# **Table S1. Adjusted means comparisons from linear mixed model excluding implausible values for energy intake, n=319 at T0, n=310 at T1 and n=246 at T2**

|  | **Diff (T1-T0)**  **95% CI** | **Diff (T2-T0)**  **95% CI** | **Diff_int_-Diff_ctr_**  **(T1-T0)**  **95% CI** | **Diff_int_-Diff_ctr_**  **(T2-T0)**  **95% CI** |
| --- | --- | --- | --- | --- |
| **Meat consumption,** g/day  Intervention  Control | **-63 [-72; -53]**  **-19 [-28; -9]** | **-42 [-54; -30]**  **-27 [-39; -15]** | **-44 [-57; -31]** | -15 [-32; 2.3] |
| **sPNNS-GS2,** [-17 to 11.5]  Intervention  Control | **1.1 [0.7; 1.4]**  **0.7 [0.3; 1.1]** | **0.6 [0.1; 1.0]**  **0.5 [0.1; 0.9]** | 0.4 [-0.2; 0.9] | 0.1 [-0.5; 0.6] |
| **GHGE,** kgCO_2_eq/day  Intervention  Control | **-1.2 [-1.5; -1.0]**  **-0.5 [-0.8; -0.3]** | **-1.4 [-1.7; -1.1]**  **-0.8 [-1.1; -0.4]** | **-0.7 [-1.1; -0.3]** | **-0.7 [-1.1; -0.2]** |
| **Energy,** kcal/day  Intervention  Control | **-250 [-328; -172]**  **-180 [-260; -101]** | **-348 [-461; -235]**  **-217 [-331; -104]** | -70 [-180; 42] | -131 [-290; 29] |

In bold: significant differences at alpha=0.05. In red: different from main analyses
